# Hope-based intervention for individuals susceptible to colorectal cancer: a pilot study

**DOI:** 10.1007/s10689-012-9545-3

**Published:** 2012-06-30

**Authors:** Samuel Mun-yin Ho, Judy Wai-chu Ho, Barbara Ka-yan Pau, Bryant Pui-hung Hui, Rosa Sze-mun Wong, Annie Tsz-wai Chu

**Affiliations:** 1 Department of Applied Social Studies, City University of Hong Kong, Tat Chee Avenue, Kowloon Tong, Kowloon Hong Kong; 2 Department of Surgery, The University of Hong Kong, Queen Mary Hospital, Pokfulam Road, Pokfulam, Hong Kong; 3 Hereditary Gastrointestinal Cancer Registry, Department of Surgery, Queen Mary Hospital, Pokfulam Road, Pokfulam, Hong Kong

**Keywords:** Hereditary colorectal cancer, Genetic testing, Hope, Psychological intervention

## Abstract

Individuals undergoing genetic testing for hereditary colorectal cancer (HCRC) are prone to develop psychological problems. This study investigated the short-term efficacy of a hope-based intervention program in increasing hope levels and decreasing psychopathology among HCRC genetic testing recipients. A longitudinal study was carried out on HCRC genetic testing recipients recruited by the Hereditary Gastrointestinal Cancer Registry. Participants joined a hope-based intervention program consisting of six sessions of weekly closed group therapy. Psychological questionnaires were administered immediately before the first and after the last sessions of the program measuring hope, anxiety and depression levels of the participants. There were 22 participants (7 men and 15 women) at a mean age of 49.4 ± 9.6 years. Women tended to have higher level of anxiety than men at pre-intervention. Paired sample *t* tests were conducted. Hope levels increased significantly from pre- to post-intervention (pre-total hope score = 5.56; post-total hope score = 6.07; *t*(1) = −0.281, *p* < 0.05). Anxiety level also decreased significantly from pre- to post-intervention (pre-anxiety score = 7.38; post-anxiety score = 5.90; *t* (1) = 2.35, *p* < 0.05). Our findings imply that hope-based intervention program would be effective in enhancing hope in HCRC genetic testing recipients. The program may also be more effective in alleviating anxiety than depression in these individuals.

## Background

Colorectal cancer (CRC) is emerging as a major health problem in Hong Kong with increasing incidence in the past decade. CRC has become the second most common and fatal cancer in Hong Kong with more than 4,335 new cases and 1,752 deaths in 2009 [1].

Previous local studies have shown Hong Kong to have an exceptionally high incidence of CRC in the young population compared with Caucasians, which is likely due to genetic causes [2, 3]. According to data from the international literature, 5–10 % of all CRC are estimated to be hereditary in nature [4]. The two main hereditary colorectal cancer (HCRC) syndromes are Familial Adenomatous Polyposis (FAP) and Hereditary Non-Polyposis Colorectal Cancer (HNPCC). Members of these families will have a 50 % chance of inheriting the mutated gene if they are the offspring of carrier and be predisposed to colorectal and related cancers, usually at young age. Predictive genetic testing is now possible for members of affected families to determine whether they have inherited the condition well before they develop the disease phenotype. Since its inception in 1995, the Hereditary Gastrointestinal Cancer Registry (the Registry), based at Queen Mary Hospital, has been offering comprehensive management for HCRC families in Hong Kong, which include genetic testing, clinical surveillance and psychosocial support.

### Psychological reactions to HCRC

Literature has shown that anyone confronted with a major illness, such as cancer, may experience disturbances in various functional domains (physical, emotional, cognitive and social) for a period of time [5]. The complicated nature of HCRC (colorectal and related cancers which may occur more than once) and the medical intervention required (including invasive preventive surgery and/or stringent lifelong surveillance program) may further amplify an individual’s psychological responses upon clinical diagnosis. Furthermore, the disease does not end with an index patient because the mutated gene can be passed on from generation to generation. Therefore, it is not difficult to envisage the enormous burden these families may have to endure.

Previous psychosocial studies on HCRC have contributed to the identification of immediate psychological sequelae relating to genetic testing of this condition [6, 7]. Other than affecting quality of life in general, these adverse psychological responses might affect and hinder individuals’ compliance to medical services [8]. These existing studies have provided strong evidence that living with HCRC (including undergoing genetic testing for this condition) is a taxing experience which may benefit from psychological intervention. However, there is a lack of theory-driven and comprehensive interventions specially designed to meet the unique needs of this group of individuals.

The Hereditary Gastrointestinal Cancer Registry in Hong Kong has been studying the psychological impact of genetic testing and its results on local HCRC families since 2001. Our previous studies have shown that HCRC patients had to endure different sorts of psychological turmoil. In one of our studies, psychological problems were found to be common among individuals undergoing genetic testing—11.9 and 20.5 % of them suffered from anxiety and depression, respectively [9]. In another study among 62 HCRC genetic testing recipients, it was found that subjects with higher depression level tended to focus more on the negative consequences of learning genetic testing results, and hence may choose to decline genetic testing [10]. There is a need to identify factors that contribute to the development of resilience which will promote positive adjustment to HCRC genetic testing.

### The role of hopefulness in HCRC genetic testing

In recent years, allied health professionals have begun to recognize that maintenance of hope is important for patients with chronic illnesses and has a significant impact on their overall physical and mental health. The model proposed by Snyder is one of the most researched theories on hope. According to Snyder [11], hope is “goal-directed thinking” in which individuals appraise their capability of producing workable routes to attain goals (pathways) and their potential to initiate and sustain movement via the pathway(s) (agency). In other words, being hopeful means believing one can set meaningful goals, figure out how to achieve them and motivate oneself to accomplish them.

Snyder’s theory has proven applicable to both clinical and social situations. Studies on chronic illness patients (including cancer patients) showed that higher dispositional hope was related to (1) greater psychological well-being and fewer psychological problems; (2) more adaptive physical health outcomes such as better health knowledge; (3) adoption of preventive health behaviors; and (4) better adjustment to chronic illnesses and pain tolerance [12–14]. In social situations, those with higher hope would show (1) better social functioning and less loneliness and (2) better performance and accomplishment in academic and athletic arenas [15]. Snyder’s hope model proposes that, when confronted with negative outcomes, such as diagnosis of a major illness or a positive genetic testing result, high-hope individuals will only be distressed temporarily but will rebound with energy and ideas for achieving life goals [16]. Our previous study has shown that high hope individuals, when compared to their low-hope counterparts, tended to exhibit more resilience throughout the HCRC genetic testing process [17]. Uncertainties in life (such as who in the family is/are affected and when one would develop cancer) and the associated distress hamper individuals in adopting positive and hopeful attitudes towards their lives, and in planning important personal and life goals including marriage and procreation. High hope individuals would probably be able to handle the above challenges better, leading to higher resilience throughout the HCRC genetic testing process.

If hopefulness is important in maintaining participants’ mental well-being throughout the HCRC genetic testing process, our next clinical task is to provide interventions to attempt to increase hope levels of our participants. Based on Snyder’s theory, the research team has designed a structured hope-based intervention program to help individuals from HCRC families to set realistic goals in life, to learn ways to motivate themselves to achieve their goals and to get on with life productively while living with their illnesses. To the best of our knowledge, this is the first hope-based intervention tailor-made for individuals with HCRC syndromes. The aim of this study was to investigate the efficacy of the hope based intervention in increasing hope levels as well as decreasing psychopathology among HCRC genetic testing recipients.

## Methods

### Participants and procedures

Ethics approval was obtained from the Institutional Review Board of the Hospital Authority Hong Kong West Cluster. Twenty-two HCRC subjects were recruited to participate in the program. There were 7 men (31.8 %) and 15 women (68.2 %). Mean age at intervention was 49.4 years (±9.6 years). For those with a cancer history (n = 9), the mean interval from first cancer diagnosis to intervention was 125.4 months (±90.1 months). A participant profile is provided in Table 1 and medical data of the participants is provided in Table 2.

**Table 1:** Participant profile (N = 22)

	n	%
Age		
30–39	3	13.6
40–49	8	36.4
50–59	8	36.4
60–69	3	13.6
Marital status		
Married	16	72.7
Single	4	18.2
Windowed	2	9.1
With children		
Yes	5	22.7
No	17	77.3
Educational level		
Primary	3	13.6
Secondary	13	59.1
Tertiary	4	18.2
No formal education/others	2	9.1
Employment status		
Worked full-time	13	59.1
Worked part-time	3	13.6
Fulltime house keeper	6	27.3

**Table 2:** Medical data (N = 22)

	n	%
Syndrome type		
FAP	14	63.6
HNPCC	8	36.4
Method of syndrome diagnosis		
Clinical diagnosis only	6	27.3
Genetic diagnosis only	7	31.8
Clinical and genetic diagnosis	9	40.9
Genetic status at the time of intervention		
Mutated gene carrier	15	68.2
Non-carrier (genetically normal)	1	4.5
Pending genetic testing result	1	4.5
Genetic testing uninformative or not applicable	5	22.7
Personal history of cancer		
Yes	9	40.9
No	13	59.1

The intervention was conducted by a clinical psychologist who had been working with the Registry on a part-time basis for 9 months before the start of the study. This facilitator had also received training on hope-based theory at the Positive Psychology Laboratory of the University of Hong Kong. The intervention program was held in a meeting room at the Cancer Center of Queen Mary Hospital. Privacy was ensured to encourage free discussion and sharing during the group sessions. Participants completed a package of questionnaires immediately before the first (pre-intervention) and immediately after the last (post-intervention) session.

### The hope-based intervention

The whole program consisted of six sessions of weekly closed group therapy based on a manual. Each session was carried out using a combination of group discussion, group exercise and home assignment. Each session lasted for 60–90 min. A manual (both in English and Chinese) has been developed by our multidisciplinary team based on Snyder’s hope theory and the team’s extensive experience in working with HCRC family members in Hong Kong. The team members consisted of a clinical psychologist who is a Professor from the City University of Hong Kong, a clinician (colorectal surgeon) who is one of the founders of the Registry and a clinical psychologist employed by the Registry. There were six chapters in the manual; one to be used for each group session. The overview and the main content of the manual/group session are illustrated in Table 3.

**Table 3:** Outline of the hope-based intervention

Session	Theme	Content
1	Introduction of the hope theory	Participants get to know each other
		Sharing of personal and medical background
		Introduction of the group: aim, focus and expectations
		Basic components of the hope theory
		Class discussion and take-home assignment
2	The setting of realistic and meaningful goals	What are realistic goals
		How to derive meaningful and realistic goals
		Class discussion and take-home assignment
3	Hope pathways—skills and strategies to achieve goals	Problem-solving skills
		Class discussion and take-home assignment
4	Hope pathways	Characteristics of people with high pathways
		How to boost one’s pathways
		Class discussion and take-home assignment
5	Hope agency—energy and motivation to achieve goals	Characteristics of people with high agency
		How to boost one’s agency
		Positive self-talk
		Class discussion and take-home assignment
6	Conclusion	Summary of the previous sessions
		Class discussion and take-home assignment
		Course evaluation

Each session had a strong emphasis on group discussion as well as on sharing and feedback among participants. Case scenarios (called hope stories) were used to stimulate discussion and strengthen the hope theories being conveyed. Apart from addressing concerns related to HCRC, specific psychological skills (such as setting realistic goals, problem-solving skills and positive self-talk) were taught and practiced in a group environment to enhance positive psychological outcome.

### Measures

#### Dispositional hope

The 12-item Adult Trait Hope Scale was rated on the basis of an 8-point Likert scale (1 = definitely false to 8 = definitely true) used to measure hope according to the model of Snyder et al. [18]. A Hope Total score was obtained by aggregating the scores for the 12 items. A Hope Pathway and a Hope Agency score were computed by summing the relevant items. The Cronbach’s reliability alphas for the Hope Total scores at T1 and T2 were 0.85 and 0.92, respectively. Both Hope Pathway and Hope Agency scores had satisfactory internal reliability alpha values according to the present sample (Hope Pathway: T1 = 0.88, T2 = 0.88; Hope Agency: T1 = 0.60, T2 = 0.88).

#### Anxiety and depression

The 14-item Chinese version of the Hospital Anxiety and Depression Scale was used to indicate negative emotions [19]. Two scores—HADS Anxiety and HADS Depression—were derived from the questionnaire. Severity of symptoms was rated according to a 4-point Likert scale. Higher scores corresponded to more symptoms of anxiety and depression, respectively. The 7/8 normative cut-off points for HADS Anxiety and HADS Depression were used to classify participants into low (a score of below or equals to 7) or high (a score of above or equals to 8) anxiety and depression, respectively [19]. For the present sample, the Cronbach’s reliability alpha values for the HADS Anxiety and HADS Depression were satisfactory (HADS Anxiety: T1 = 0.89 and T2 = 0.90; HADS Depression: T1 = 0.69 and T2 = 0.74).

## Results

### Descriptive statistics

The mean and standard deviation of the psychological variables are shown in Table 4. Independent sample *t* tests showed that women, compared to men, tended to have higher level of anxiety at pre-intervention. No difference was observed in other medical and demographic variables. Gender was controlled in subsequent analyses.

**Table 4:** Mean and standard deviation of variables by gender: pre-intervention (time 0)

	Gender		*t* value
	Male (n = 7) Mean (SD)	Female (n = 15) Mean (SD)	
HADS anxiety	4.43 (3.26)	8.47 (4.39)	2.16*
HADS depression	4.00 (2.38)	6.00 (3.42)	1.39
Hope pathway	5.71 (2.03)	5.72 (1.08)	0.004
Hope agency	5.54 (1.37)	5.65 (1.18)	0.20
Hope total	5.62 (1.57)	5.68 (1.07)	0.10

* *p* < 0.05

### Correlations between hope and anxiety and depression

Partial correlations controlling for gender were conducted at the pre- and post-intervention levels. The hope pathway score was negatively correlated with anxiety and depression levels both at the pre- and post-intervention time points. This result showed that participants with more strategies to achieve their goals tended to exhibit less anxiety and depression symptoms (Table 5). In general, total hope showed similar patterns with the exception that total hope was not correlated with anxiety level at pre-intervention.

**Table 5:** Partial correlations controlling for gender as a covariate (n = 22)

	HADS anxiety	HADS depression	Hope pathway	Hope agency	Hope total
HADS anxiety	1	0.80**	−0.59**	−0.36	−0.51*
HADS depression	0.53*	1	−0.47*	−0.38	−0.45^a^
Hope pathway	−**0.38**	−**0.40**	1	0.74	0.94
Hope agency	−**0.37**	−0.64**	0.72**	1	0.92
Hope total	−**0.40**	−0.55*	0.94**	0.92**	1

Pre-intervention correlation coefficients are presented in the lower triangle in bold fronts. Post-intervention correlation coefficients are presented in the upper triangle

* *p* < 0.05; ** *p* < 0.01; ^a^ *p* = 0.051

### Levels of hope over time

A major objective of this study is to investigate whether our hope-based intervention program can increase hope levels of the participants within a 6-week period. Paired sample *t* tests were conducted to examine the pre- and post-intervention change. Our results showed that all scores of the hope scale increased significantly after the intervention. Furthermore, anxiety levels of the participants decreased significantly after the intervention (Table 6).

**Table 6:** Pre- and post-intervention comparison: paired sample *t* test (n = 22)

	Pre-intervention mean (SD)	Post-intervention mean (SD)	*t* value
HADS anxiety	7.38 (4.43)	5.90 (4.55)	2.35*
HADS depression	5.52 (3.20)	4.71 (3.17)	1.47
Hope pathway	5.65 (1.42)	6.11 (1.54)	−2.30*
Hope agency	5.46 (1.17)	6.02 (1.36)	−2.88*
Hope total	5.56 (1.21)	6.07 (1.35)	−0.281*

* *p* < 0.05

### Changes in caseness for anxiety and depression as a result of the intervention

Similar to our previous study [17], we used the 7/8 cut-off of the HADS to classify each subject at each time point as a case (with a score ≥8) or a non-case (with a score ≤7) [19]. For example, if a subject had a HADS Anxiety score of 9 at pre-intervention, then he or she was classified as a HADS Anxiety case. If the same subject had a HADS Anxiety score of 3 at post-intervention, then he/she was considered to be a HADS Anxiety non-case. Table 7 shows the change in category of the participants. For anxiety, four participants changed from HADS Anxiety case to non-case after the intervention, and the percentage of change was significant (χ^2^(1) = 9.96, *p* < 0.01). For depression, again four participants changed from HADS Depression case to non-case. However, it should also be noted that 2 participants changed from non-case to case after the intervention. In other words, two participants became more depressed after the intervention. Nonetheless the decrease in depression caseness was statistically significant.

**Table 7:** Pre- and post-intervention change in depression and anxiety case (7/8 cutoff)

	Post-intervention case	Post-intervention non-case	χ^2^(1) value
HADS anxiety			
Pre-intervention case	9	4	9.69**
Pre-intervention non-case	0	8	
HADS depression			
Pre-intervention case	7	4	4.073*
Pre-intervention non-case	2	8	

One participant had missing values in the Hospital Anxiety and Depression Scale

* *p* < 0.05; ** *p* < 0.01

## Discussion

This paper reports the authors’ effort in establishing a group psychotherapy program that may help HCRC family members to set realistic goals in life, to learn ways to motivate themselves in achieving their goals and to get on with life productively while living with their illnesses.

Unlike sporadic form of colorectal cancer, HCRC is a lifelong illness—an affected individual may develop cancer more than once in his/her lifetime. HCRC is also a family illness—more than one family member may be affected and the offspring of an affected individual will have a 50 % chance of inheriting the condition, and if found to be a carrier will have 80–100 % chance of developing clinical illness some time in his/her life. The uncertainties and adversities imposed by this condition result in untoward psychological burdens in HCRC family members. Our previous study confirmed that a significant proportion of these individuals had clinically significant psychological symptoms [9]. Furthermore, our clinical experiences informed us that, even for those without clinical depression or anxiety, these individuals had a pessimistic outlook on life and experienced difficulties in formulating life goals. In Snyder’s terms, these individuals are low on both pathways and agency.

One of the strengths of Snyder’s hope theory is that anyone, of whatever background, can acquire the skills to improve his/her pathways and agency; and with proper training, a person can regain hopeful attitudes towards life. Attracted by the applicability of Snyder’s theory to both clinical and general populations, the authors began investigating the use of a hope-based group intervention therapy on HCRC family members. Based on a manual for a hope-based intervention for adult provided by McDermont and Snyder [20], a manual was developed by our team following the same theoretical framework and psychological skills used by Snyder and supplementing these with locally applicable and HCRC-relevant hope-based stories.

Our results showed that a 6-week systematic hope-based intervention program could increase the hope levels of our participants. Our findings showed that participants became more hopeful overall while also exhibiting increases in pathways and agency components of hope. Furthermore, the anxiety levels of our participants significantly decreased from pre- to post-intervention. Participants’ depression level did not significantly decrease from pre-to post-intervention and some individuals changed from depression non-case to case. In our study, a hope-based intervention program tended to be more effective in alleviating anxiety than reducing depression. Given that anxiety is a key symptom of HCRC genetic testing recipients [17]. The effectiveness of hope-base intervention program in reducing anxiety may enhance the well-being of HCRC genetic testing recipients.

This study has several limitations. The sample size of the study was relatively small; hence our findings can be generalized only with caution. Moreover, we did not follow the participants after the six sessions of hope-based intervention program. Future research may study the long term effect of such program. Studies involving larger sample sizes and a randomized controlled trial design are now needed to further demonstrate the efficacy of the intervention.

## Conclusions

This was the first hope-based intervention program for HCRC genetic test recipients. The program was effective in enhancing hope level and reducing anxiety of the participants. Future study should be conducted to examine the long term effect of such program on the psychological health of HCRC genetic testing recipients.
